# Schwannoma in the Midline of Hard Palate: A Case Report and Review of Literature

**DOI:** 10.5681/joddd.2014.021

**Published:** 2014-06-11

**Authors:** Monir Moradzadeh Khiavi, Ali Taghavi Zenouz, Ali Hossein Mesgarzadeh, Omid Sabetmehr, Seyyed Mostafa Mahmoudi, Maryam Kouhsoltani

**Affiliations:** ^1^Associate Professor, Departments of Oral Pathology, Faculty of Dentistry, Tehran University of Medical Sciences, International Campus, Tehran, Iran; ^2^Associate Professor, Department of Oral Medicine, Faculty of Dentistry, Tabriz University of Medical Sciences, Tabriz, Iran; ^3^Associate Professor, Department of Oral and Maxillofacial Surgery, Faculty of Dentistry, Tabriz University of Medical Sciences, Tabriz, Iran; ^4^Assistant Professor, Department of Oral and Maxillofacial Surgery, AJA University of Medical Sciences, Tehran, Iran; ^5^Assistant Professor, Department of Oral Pathology, Faculty of Dentistry, Birjand University of Medical Sciences, Birjand, Iran; ^6^Assistant Professor, Department of Oral Pathology, Faculty of Dentistry, Tabriz University of Medical Sciences, Tabriz, Iran

**Keywords:** Palate, oral cavity, schwannoma

## Abstract

Schwannoma is a benign encapsulated slow-growing tumor that originates from Schwann cells of the peripheral nerve sheath. It usually occurs in the head and neck; however, it is rare in the oral cavity. The tongue is the most common site of intraoral schwannomas, followed by the floor of the mouth, palate, gingiva, vestibular mucosa, lips and mental nerve area. We report a rare case of schwannoma in the midline of hard palate with ulcerated surface in a 21-year-old male with a two-month history of a painless swelling on his palate. Clinical, radiographic and histopathological features along with differential diagnosis and treatment are also discussed.

## Introduction

Schwannoma, also known as neurilemoma, neurinoma and Schwann cell tumor is a benign tumor that originates from perineural Schwann cells of the nerve sheath.^1^ Approximately 25-45% of the lesions occur in the head and neck region;^1-3,4^ however, intraoral lesions are rare.^1,5^ The most common intraoral site is the tongue, followed by the floor of the mouth, palate, gingiva, vestibular mucosa, lips and mental nerve area.^1,2^ Biswas et al reviewed a series of 31 cases of extracranial head and neck schwannomas during a 10-year period. Only one case was in the hard palate.^6^

Schwannoma is usually an encapsulated slow-growing painless solitary lesion with a smooth surface.^1,7,8^It can occur at any age but it most commonly arises in the second and third decades of life.^8^ It is unclear whether schwannoma has a predilection for women, men, or occurs in both sexes equally.^8,9^The etiology of schwannoma is unknown.^1^The lesions such as fibroma, lipoma, neurofibroma and salivary gland tumors can be included in the clinical differential diagnosis of hard palate schwannoma.^9^The aim of this case report is to present a schwannoma in the midline of the palate which is an unusual location for intraoral tumors.

## Case Report

**Figure 1.**
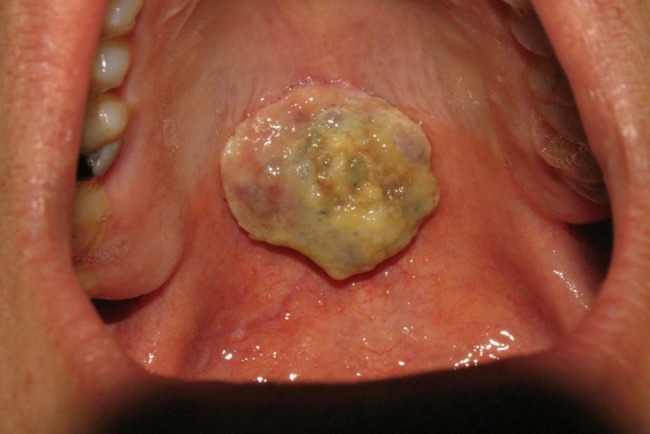
The schwannoma presented as a pedunculated mass in the midline of hard palate with an ulcerated yellowish surface.

**Figure 2.**
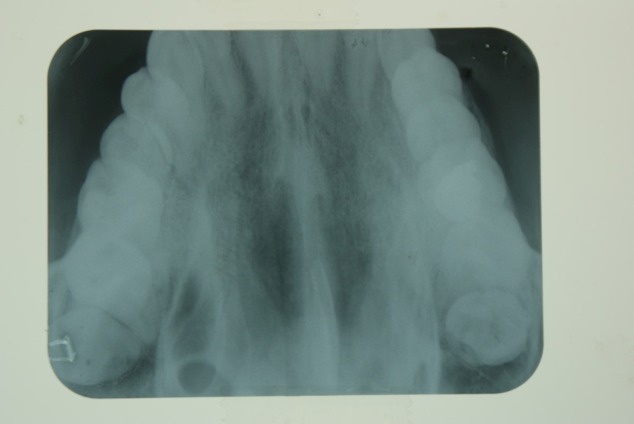
There is no osseous alteration on the occlusal radiograph.

**Figure 3.**
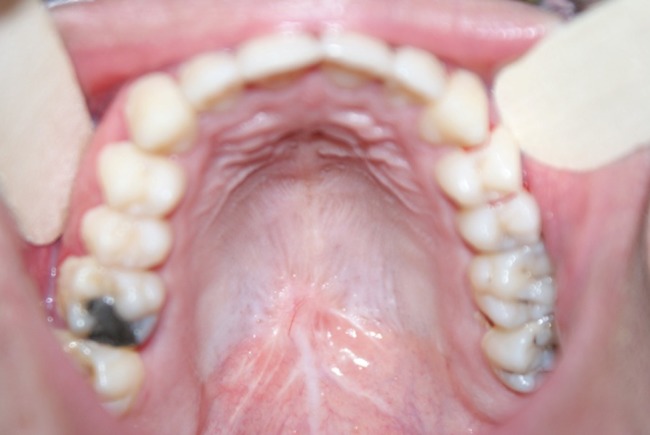
Palisading spindle cells surrounding central eosinophilic areas (Antoni A) as well as less cellular and less organized area (Antoni B) (H&E, original magnification ×200, inset ×400 ).

**Figure 4.**
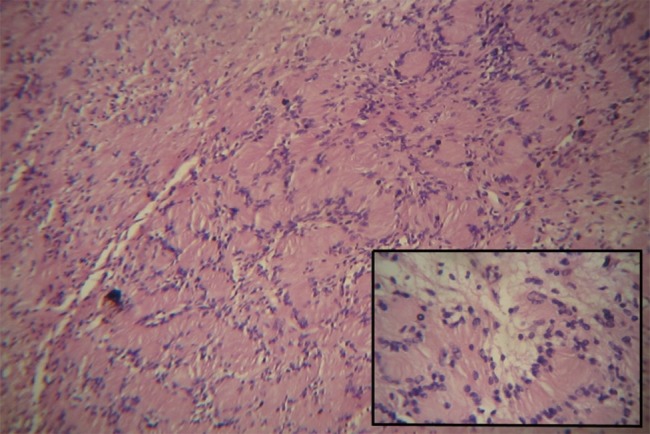
Immunostaining of tumoral cells that were strongly positive for S-100 (original magnification ×400).

**Figure 5.**
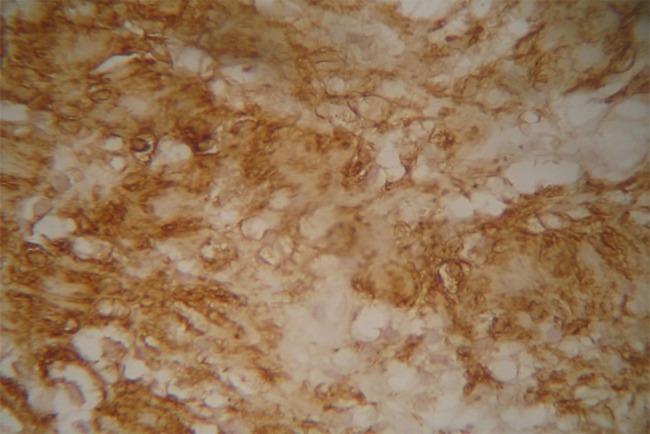
After 6 months the patient is without recurrence.

A 21-year-old male referred to the Faculty of Dentistry, Tabriz University of Medical Sciences, with a two-month history of an asymptomatic mass in his palate. He had no history of systemic diseases. Extraoral examination revealed no significant signs. There were no palpable lymph nodes. Intraoral examinations revealed a 2×2-cm pedunculated mass in the midline of the palate. The lesion was non-tender and firm in consistency and had an ulcerated yellowish surface in most areas (Figure 1). There were no osseous alterations on occlusal radiographs (Figure 2). Salivary gland tumors and benign mesanchymal lesions were included in the differential diagnosis. Incisional biopsy was performed under local anesthesia. Histopathological evaluation showed proliferation of spindle-shaped cells with palisaded arrangements around the central acellular area in most parts. Areas of less cellularity and less organized portions were also observed (Figure 3). The overlying epithelium had been replaced by a finbrinopurulent membrane. The results of imunohistochemical staining for S-100 protein were positive (Figure 4). According to histopothological and immunohistochemical findings the diagnosis was schwannoma. After one week, complete excision of the lesion was carried out under general anesthesia and the final histopathological diagnosis was schwannoma, too. After 6 months of follow-up there was no recurrence of the lesion (Figure 5).

## Discussion

Schwannoma is a benign, slow-growing, and usually solitary encapsulated tumor that originates from Schwann cells of the peripheral nerve sheath.^1,5,9,10^It is more prevalent in head, neck and surface flexors of the upper and lower extremities. However, intraoral lesions are infrequent.^1^ The most common location of intraoral schwannoma is the tongue and it rarely occurs in the hard palate.^1,4^ It was reported in 1987 by Jones for the first time at this site. So far 16 cases of palatal schwannoma have been reported in the English literature, which have been summarized in Table 1. Among the reported cases females have been affected more than males;^4,5,10,14,15^ however, this case was observed in a male patient. Gender distribution of tumor in various studies is different. William et al showed that schwannomas have a predilection for males, while in the study of Lucas, there was a greater predilection for females, and Hatziotis and Asprides, and Enzinger and Weissreported an equal distribution between both sexes;^4,8^however, there is a high tendency for female among the reported cases.^4,5,07,10-15^

**Table 1:** Reports of schowannomas of palate published in the literature

Authors	Year	Sex	Age	Location	Duration
Jones^11^	1987	F	29	Hard & soft palate	2 years
Krolls^12^	1994	F	21	Hard palate	3 years
Amir^17^	2002	M	40	Hard palate	3 Months
Rabbels^13^	2005	F	11	Hard Palate	3 Months
Lopez-Carriches^1^	2009	Unknown	15	Hard palate	3 Months
Ashok Murthy^15^	2009	F	28	Hard palate	4 Months
Lollar^2^	2010	M	33	Hard palate	3 Months
Santos^7^	2010	F	41	Right hard palate	5 years
Santos^7^	2010	F	53	Hard palate	6 Months
Isildak^4^	2010	F	45	Hard palate	15 years
Dhupar^18^	2012	M	10	Hard palate	5 Months
Santos^14^	2011	F	3	Hard palate	6 Months
Chawla^16^	2011	M	9	Soft palate	Unknown
Rahpeyma^5^	2012	F	12	Soft palate	3 Months
Shetty^10^	2012	F	70	Right hard palate	2 years
Kumar^9^	2012	M	18	Left hard palate	Unknown
Present case	—	M	21	Hard palate	2 months
The data in the table has been populated based on a search in relevant articles published in English.					

Schwannoma can occur at any age but they most commonly occur in the second and third decades of life.^8^ Age distribution of the reported cases range from 3 to 70 years of age^1,2,4,5,7,9-18^ and the peak age is the second decade of life. The majority of palatal schwannomas have been reported on the lateral aspect of the palate^1,7,9-14,15^ Schwannomas are usually solitary lesions; however, in rare cases they can be multiple as a sign of von-Recklinghausen's neurofibromatosis.^19^

Although ulceration of the overlying epithelium is rare,^19^ in our case the epithelum was ulcerated and replaced by a yellowish membrane.

Although schowannoma is a painless lesion, the pressure of the tumor on an adjacent nerve may cause paresthesia.^5^However, there was no pain or paresthesia in the present case.

The clinical differential diagnosis of a slow-growing lesion in this region is more likely a salivary gland lesion, including benign and low-grade malignant salivary gland tumors and also less mesenchymal lesions, including benign and a low-grade malignant neoplasm of mesenchymal origin.^20^In our case the differential diagnosis list included salivary gland tumors and also benign mesanchymal tumors.

Among benign salivary gland tumors, the pleomorphic adenoma is the most probable lesion to occur in this region. The palate is the most common site for minor salivary gland pleomorphic adenomas. Palatal tumors present as a painless slow-growing swelling with smooth surface that can be ulcerated due to trauma.^20-22^

Among the malignant salivary neoplasms, mucoepidermoid carcinoma would be the most possible lesion. Mucoepidermoid carcinoma is the most common malignant salivary gland neoplasm. After parotid, the minor salivary glands constitute the second most common site for mucuepidermoid carcinoma, especially the palate. Clinically, mucoepidermoid carcinoma appears as an asymptomatic swelling.^20,22,23^

Schwannomas exhibit two microscopic patterns in varying amounts: Antoni A and Antoni B. Streaming fascicles of spindle-shaped Schwann cells are characterized by Antoni A. These cells often form a palisaded arrangement around central acellular eosinophilic areas known as Verocay bodies. Antoni B tissue is less cellular and less organized.^10^In this case, Antoni A was more prominent than Antoni B. For definitive diagnosis, immunostaining analysis for S-100 is necessary.^8^

Degenerative changes can be seen in some older tumors that are known as ancient schannoma. Ancient schwannoma is reported in the oral cavity.^3^

Schwannoma is treated by surgical excision. After treatment the lesion usually does not recur and malignant transformation is extremely rare.^9,19^

## Conclusion

Schwannoma is a slow-growing benign tumor that is rare in the hard palate. It is difficult to diagnose this tumor based on clinical appearance; as a result, histopathological examination is necessary for a definite diagnosis.
